# Cephalosporins inhibit human metallo β-lactamase fold DNA repair nucleases SNM1A and SNM1B/apollo†
†Electronic supplementary information (ESI) available: General experimental procedures and supplementary figures. See DOI: 10.1039/c6cc00529b



**DOI:** 10.1039/c6cc00529b

**Published:** 2016-04-18

**Authors:** Sook Y. Lee, Jürgen Brem, Ilaria Pettinati, Timothy D. W. Claridge, Opher Gileadi, Christopher J. Schofield, Peter J. McHugh

**Affiliations:** a Department of Oncology , Weatherall Institute of Molecular Medicine , University of Oxford , John Radcliffe Hospital , Oxford OX3 9DS , UK . Email: peter.mchugh@imm.ox.ac.uk; b Department of Chemistry , University of Oxford , 12 Mansfield Road , Oxford OX1 3TA , UK . Email: christopher.schofield@chem.ox.ac.uk; c Structural Genomics Consortium , Old Road Campus Research Building , Roosevelt Drive , University of Oxford , OX3 7DQ , UK

## Abstract

Bacterial metallo-β-lactamases (MBLs) are involved in resistance to β-lactam antibiotics including cephalosporins. Human SNM1A and SNM1B are MBL superfamily exonucleases that play a key role in the repair of DNA interstrand cross-links, which are induced by antitumour chemotherapeutics, and are therefore targets for cancer chemosensitization. We report that cephalosporins are competitive inhibitors of SNM1A and SNM1B exonuclease activity; both the intact β-lactam and their hydrolysed products are active. This discovery provides a lead for the development of potent and selective SNM1A and SNM1B inhibitors.

## Abstract 2

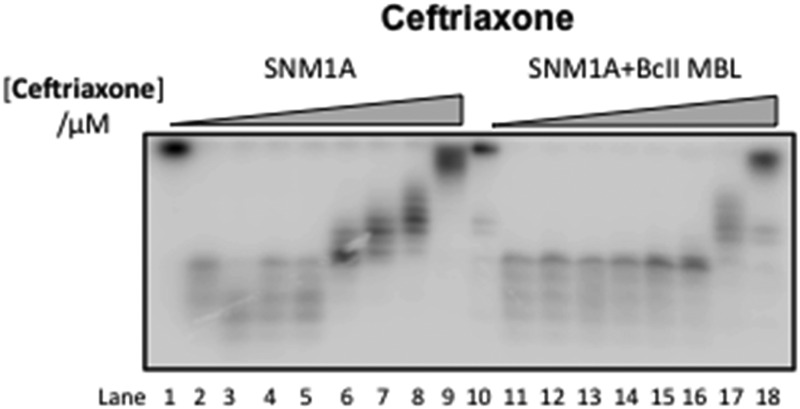
The human metallo-β-lactamase fold DNA repair enzymes SNM1A and SNM1B can be inhibited by cephalosporin-family molecules, revealing a potential new strategy to chemosensitive tumours, as well as a novel evolutionary link.

β-Lactamases are highly efficient enzymes that enable bacterial resistance to the clinically important β-lactam antibiotics. β-Lactamases are divided into those employing a nucleophilic serine residue in catalysis (Class A, C and D) and those employing zinc ions, the metallo β-lactamases (MBLs).^1^ While clinically useful inhibitors of the serine-β-lactamases have been developed, this is not the case for the MBLs, which represent a clinical threat because they catalyze the hydrolysis of almost all types of β-lactam antibiotic (Scheme 1A), including pencillins, carbapenems and cephalosporins, with the monobactams being an exception.^2–5^

**Scheme 1:**
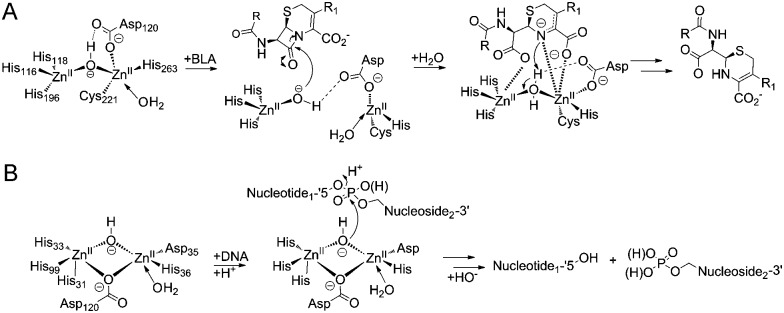
(A) Reaction scheme showing an outline mechanism for the hydrolysis of β-lactam antibiotic by MBLs. (B) Reaction scheme showing the hydrolysis of a phosphodiester bond by SNM1B.

Pioneering structural studies on the MBL from *Bacillus cereus* (BcII) revealed an unanticipated fold comprising two anti-parallel β-sheets flanked by α-helices with metal-binding sites at the edge of the β-sandwich (Fig. 1A).^6^ This fold supports binding of one or two zinc ions depending on the MBL subclass (B1 and B3 MBLs are di-Zn(ii) enzymes, whereas B2 MBLs are normally mono-Zn(ii) enzymes) (Fig. 1D).^1,7^ The active site of the MBL-fold proteins contains five conserved motifs, which are involved in zinc ion binding and/or catalysis.^1,8^ In the light of structural studies with the BcII MBL,^6^ multiple other MBL-fold enzymes have been identified by bioinformatics, followed by structural studies performed.^4^ In humans there are 18 assigned MBL fold proteins that are involved in diverse metabolic processes, including small molecule metabolism and nucleic acid hydrolysis.^4,8^

**Fig. 1:**
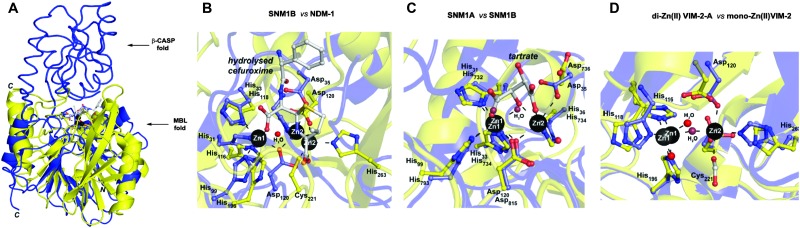
(A) Views from a crystal structure of SNM1B in blue (PDB: 5AHO) overlayed with that of NDM-1 in yellow (PDB: ; 4RL0). (B) Active site structure of SNM1B (blue) overlaid with that of NDM-1 (yellow). (C) Active site structure of SNM1A (blue) (PDB: ; 5AHR) overlaid with that of SNM1B (yellow). (D) Active site structure of di-zinc VIM-2-A (blue) (PDB: ; 1KO2) overlaid with that of mono-zinc VIM-2 (yellow) (PDB: ; 4BZ3). Key conserved residues are shown as sticks and the active site zincs are black spheres.

SNM1A and SNM1B are 5′–3′ exonucleases (Scheme 1B) that are involved in the repair of damaged DNA. They posses a highly-conserved MBL-fold domain and a β-CASP (CPSF-Artemis-SNM1-Pso2) domain; the presence of the latter is characteristic of many nucleic acid-processing MBL superfamily proteins (Fig. 1A). There are three highly conserved motifs in the β-CASP domain that are involved in the coordination of the active site zinc ions.^1,4,7–9^ SNM1A/B are important in interstrand crosslink (ICL) repair where they are involved in the lesion-uncoupling step (‘unhooking’) of ICLs. Importantly, and unusually, their exonuclease activity can resect past site-specific crosslinks.^10^ Cells depleted in SNM1A and SNM1B show increased sensitivity to ICL-inducing agents including *cis*-platin and mitomycin C (MMC),^11,12^ which are routinely used as cancer chemotherapeutics.^13^ As such, compounds that inhibit SNM1A/B could potentiate the effect of these drugs or be useful in circumventing resistance to them.

Cephalosporins are an important class of β-lactam antibacterials that are irreversible inhibitors of penicillin-binding protein (PBPs) involved in bacterial cell wall synthesis.^14^ Because, the active site architectures of bacterial MBLs and SNM1A/B are similar (Fig. 1B and C),^15^ and β-lactam antibiotics, including cephalosporins, are the targets of bacterial MBLs, we screened a panel of β-lactam antibiotics and related molecules as candidate inhibitors of SNM1A/B. Here we report that certain cephalosporins, but not penicillins, carbapenems, monobactams are reversible inhibitors of SNM1A/B.

We first screened a panel of β-lactam antibiotics (cephalosporins, penicillins and carbapenems, the only clinically used monobactam β-lactam antibiotic aztreonam and the recently introduced serine-β-lactamase inhibitor avibactam) against SNM1A/B using a modified real-time fluorescence assay (ESI,† Tables S1 and S2),^10^ which uses a 20-nucleotide single-stranded DNA substrate modified with a 5′-fluorophore, fluorescein, and a ‘Black-Hole Quencher 1’ (BHQ1) located eight nucleotides to the 3′ side of the fluorophore (Experimental in ESI†). The intact substrate does not fluoresce when excited at 495 nm; following SNM1A/B catalysed hydrolysis, the fluorescein is ‘uncoupled’ from the BHQ1 resulting in fluorescence at 515 nm. Initially, we assayed the relative (compared to a DMSO control) activity of SNM1A/B in the presence of the potential inhibitors (1 mM). Potentially active compounds (ESI,† Fig. S1) were validated using a gel-based nuclease assay employing the same substrate as used in the fluorescence-screen (Fig. 2 and ESI,† Fig. S2). Four ‘hits’, all cephalosporins, with a *z*-score of –1 or less (ESI,† Fig. S1) in the initial assays: 7-aminocephalosporinic acid, cephalosporin C, cefotaxime, and ceftriaxone – were confirmed as nuclease inhibitors using the direct nuclease assay (Fig. 2 and ESI,† Fig. S2). Their IC_50_ values were subsequently determined using the real-time fluorescence assay (Table 1), which has been validated for SNM1A/B kinetics.^10^ Despite the fact that the SNM1s possess active sites closely related to those of the bacterial MBLs, it is striking that of the β-lactams tested, only compounds with a cephalosporin, but not penicillin, carbapenem or monobactam core are active. The four cephalosporins are generally more active against SNM1A than SNM1B (Table 1); 7-ACA, cefotaxime and ceftriaxone inhibit SNM1A with IC_50_s of 4–7 μM but inhibit SNM1B with IC_50_s of 32–129 μM. Using this assay, the *K*_M_ and *V*_max_ parameters of SNM1A/B were determined in the absence and increasing concentrations of the inhibitor. Since the observed *V*_max_ was independent of the inhibitor concentration, the cephalosporins likely act as competitive reversible inhibitors of SNM1A/B (Fig. 3 and ESI,† Fig. S3). We further validated their mode of inhibition of SNM1A/B using thermal (*T*_M_) shift assays, where we observed that the melting temperature of SNM1A decreases in the presence of all the hit compounds (Table 1).

**Fig. 2:**
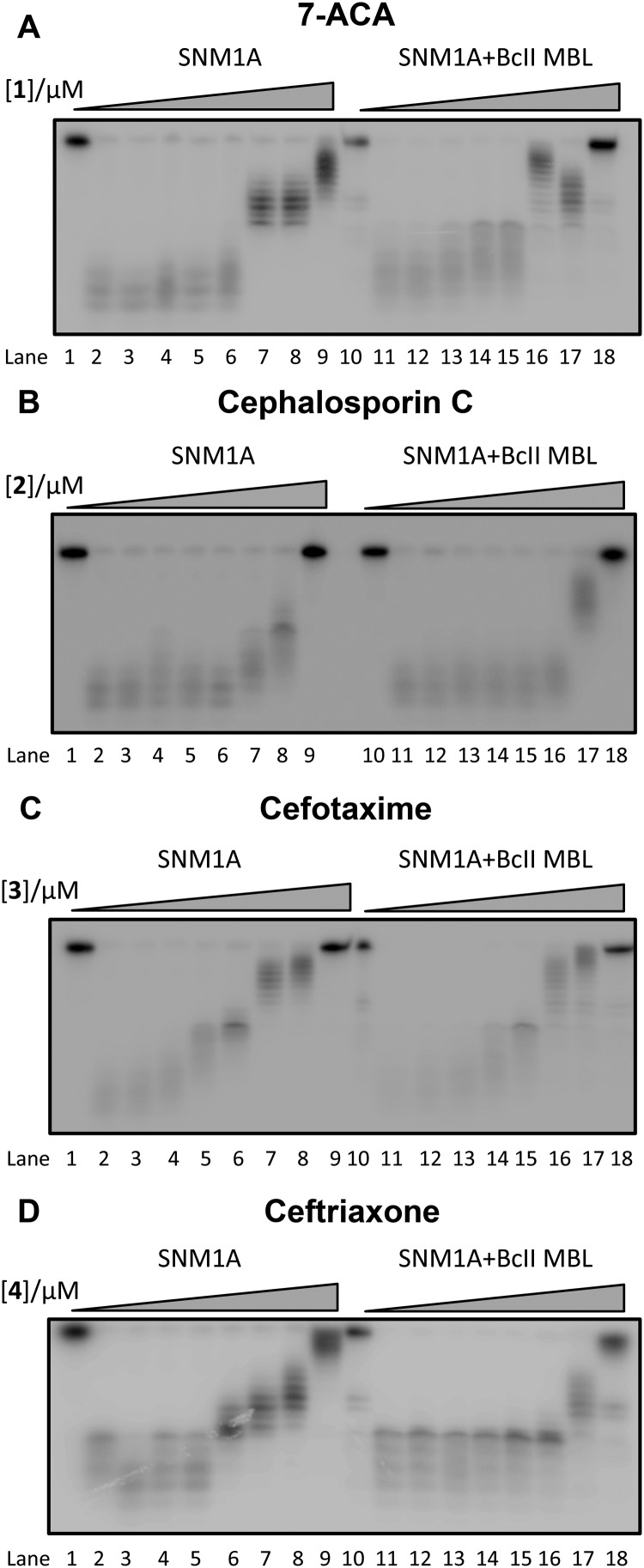
Gel Images showing the effect of increasing concentration of (A) 1 (7-ACA), (B) 2 (cephalosporin C), (C) 3 (cefotaxime), and (D) 4 (ceftriaxone) on the digestion of a 3′-radiolabelled 21 nucleotide DNA substrate (100 nM) by SNM1A (0.4 nM) in the presence (Lanes 10–18) and absence (Lanes 1–9) of 2 μM BcII MBL.

**Table 1:** Table showing IC_50_ values of the 4 hit compounds against SNM1A and SNM1B and *T*_m_ shifts of the hits with SNM1A

Name	IC_50_ SNM1A^*a*^/μM	IC_50_ SNM1B^*a*^/μM	*T*_m_/°C	*T*_m_ shift/°C
SNM1A only			56.9 ± 0.1	
7-ACA	7 ± 1	32 ± 10	41.0 ± 0.2	–15.9
Cephalosporin C	61 ± 27	>500	—	—
Cefotaxime	5 ± 2	129 ± 20	37.7 ± 0.2	–19.2
Ceftriaxone	4 ± 2	40 ± 14	37.1 ± 0.4	–19.8
Ceftazidime	>500	>500	56.7	–0.22

^*a*^Determined using Prism software from 3 independent repeats.

**Fig. 3:**
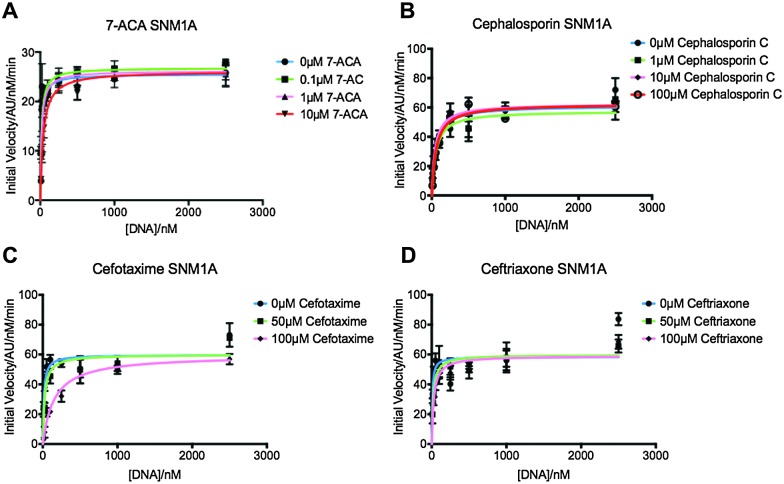
Graphs showing Michaelis–Menten curves of SNM1A in increasing concentrations of 7-ACA (A), cephalosporin C (B), cefotaxime (C) and ceftriaxone (D). Graphs fitted using Prism software and *K*_M_ and *V*_max_ values generated using these curves. Error bars generated from 3 independent repeats.

We then investigated whether SNM1A/B can catalyse hydrolysis of the cephalosporin β-lactam ring. First, we used a chromogenic cephem (nitrocefin) based hydrolysis assay to test for β-lactamase activity.^16^ Compared to the MBL from *Bacillus cereus* (BcII),^17^ SNM1A/B showed low, if any, β-lactamase activity (data not shown). However, nitrocefin does not possess a polar group on its C-7 side chain, as do the four cephalosporins identified as SNM1 inhibitors. We thus used ^1^H NMR spectroscopy (^1^H NMR, 700 MHz) to investigate whether SNM1A catalyses hydrolysis of the inhibitory cephalosporins (ESI,† Fig. S4). We used relatively high enzyme (SNM1A) concentrations of up to a 1 : 10 SNM1A : cephalosporin ratio and monitored ^1^H NMR spectra for up to 12 h. Within experimental error we did not observe enzyme-catalysed hydrolysis of the tested cephalosporins. Using similar assay conditions, when the same amount of cephalosporin was incubated with the BcII MBL using a 1 : 40 000 BcII : substrate ratio; the cephalosporins were completely hydrolysed within 10 minutes (ESI,† Fig. S4). Taken together, these results suggest that SNM1A/B do not, at least efficiently, catalyse cephalosporin hydrolysis.

We then investigated the correlation between the obtained IC_50_ values and binding of the cephalosporins. We used the ^1^H NMR based waterLOGSY technique,^18^ which has high sensitivity as compared to related NMR based techniques. In agreement with the IC_50_ values, the waterLOGSY results revealed that ceftriaxone was the best binder followed by cefotaxime, then 7-ACA (ESI,† Fig. S5).

To investigate binding of a hydrolysed product to SNM1A, the most potent inhibitor ceftriaxone was incubated with BcII. The hydrolysed cephem product was then purified; ^1^H NMR analysis revealed a lack of detectable ceftriaxone (ESI,† Fig. S6A).^19^ The results of incubations with nitrocephin indicated that no BcII was present in the purified hydrolysed ceftriaxone. waterLOGSY analysis revealed the hydrolysed ceftriaxone as a poor binder compared to the intact cephalosporins (ESI,† Fig. S6B). The combined waterLOGSY results imply the following order of binding affinity: ceftriaxone (strongest binder) > cefotaxime > 7-ACA > hydrolysed ceftriaxone (weakest binder). Quantitative ^1^H NMR binding assays^20^ were possible only for the strongest binder, *i.e.* intact ceftriaxone which manifested a *K*_d_ = 19 ± 4 μM (ESI,† Fig. S7). When the four cephalosporin inhibitors were incubated with the BcII MBL to ensure complete hydrolysis, then tested for inhibition of SNM1A and SNM1B, the products were found to inhibit similarly to the intact cephalosporins (Fig. 2 and ESI,† Fig. S2).

In summary, the results reveal that cephalosporins are competitive inhibitors of SNM1A and SNM1B, with IC_50_ values in the μM range. Interestingly, we observed that all four cephalosporin inhibitors were more potent against SNM1A than SNM1B (Table 1). Although other factors are possible, this difference may reflect the observation that the active site of SNM1A (PDB: ; 5AHR) is apparently more accessible than that of SNM1B (PDB: ; 5AHO) as indicated by crystallographic analyses.^15^ Alternatively, it may reflect different metal usage by the two nucleases.^10^ Both the intact cephalosporins and the hydrolysed ‘products’ were observed to be SNM1A and SNM1B inhibitors, though in the case of ceftriaxone, the intact compound was observed to bind more tightly. Thus, the enzyme inhibitor complexes likely predominantly contain an intact β-lactam ring, though it cannot be ruled out that the hydrolysed β-lactams are also inhibitors. Structural and mechanistic work on MBLs (and penicillin binding proteins) reveals binding of hydrolysed products to the active site.^21–23^ We did not observe evidence for SNM1A/B catalysed β-lactam hydrolysis; this may be because the intact products are inhibitors, so any enzyme-catalysis (if it occurs) would be suppressed. We have observed a similar situation with rhodanine-based compounds and their hydrolysed products which are bacterial MBL inhibitors.^3^

We did not observe evidence for SNM1A/B inhibition by the four studied cephalosporins in cells (ESI,† Fig. S8). This is likely because cephalosporins act as anti-bacterials *via* an extracellular mechanism, and most have been developed to have low membrane permeability.^24,25^ Modification to make cell-penetrating cephalosporin based SNM1A/B inhibitors is the subject of ongoing work. The results also suggest that more extensive cross-screening of MBL inhibitors/β-lactam based antibiotics may be profitable in terms of identifying leads for SNM1A/B inhibitors. In this regard, it is notable that reported results imply differences in inhibitor selectivity between the bacterial MBLs and SNM1A/B (*e.g.* some captopril seteroisomers inhibit bacterial MBLs, but do not inhibit SNM1A/B^26^). Given the critical roles of SNM1A/B in DNA repair it would also seem prudent to test new β-lactam antibiotics, especially cephalosporin-based compounds, for SNM1A/B inhibition in counter-selectivity screens.

Overall, the discovery of inhibition of SNM1A/B by cephalosporins is of interest from the perspective of identifying potent and selective inhibitors for use in target validation studies. The result also highlights an interesting chemical relationship between two enzymes families sharing common structural and active site platforms, both of which are involved in resistance to drugs of immense medicinal importance. They are also notable since it would seem that the true antibiotic resistance MBLs may have evolved from the more widely distributed and likely earlier MBL fold nuclease subfamily.

This work (PJM, CJS, OG) was supported by the Medical Research Council (MRC; MR/L007665/1) and SYL is funded by the Agency for Science, Technology and Research Singapore (A*STAR). The SGC is a registered charity (number 1097737) that receives funds from AbbVie, Bayer Pharma AG, Boehringer Ingelheim, the Canada Foundation for Innovation, Genome Canada, Janssen, Merck & Co., the Novartis Research Foundation, the Ontario Ministry of Economic Development and Innovation, Pfizer, São Paulo Research Foundation-FAPESP, Takeda, and the Wellcome Trust [092809/Z/10/Z].
