# Supplementary material for: Cephalosporins inhibit human metallo β-lactamase fold DNA repair nucleases SNM1A and SNM1B/apollo
Source: Chem Commun (Camb). 2016 Apr 18;52(40):6727–30. doi: 10.1039/c6cc00529b (PMC5063058; doi:10.1039/c6cc00529b)
Supplement: Supplementary file 1 [file CC-052-C6CC00529B-s001.pdf]

Experimental Procedures:

Protein Purification: ΔN-SNM1A (676-1040) and ΔC-SNM1B (1-335) were purified as described in Allerston et.al. (Allerston et al. 2015). In brief, ΔN-SNM1A and ΔC-SNM1B were purified from recombinant Sf9 insect cells. The insect cells were lysed by sonication (15 minutes on ice) and the cleared cell lysates loaded onto an AKTA-Express 5ml Ni-NTA (nickel-agarose beads). N-Terminally His6-tagged ΔN-SNM1A or ΔC-SNM1B was eluted with 300mM imidazole and then incubated with 1:20 by mass of TEV protease (Tobacco Etch Virus protease) (overnight, 4°C) to remove the His6-tag. The proteins were then dialysed overnight in buffer containing 10mM imidazole. The cleaved ΔN-SNM1A and ΔC-SNM1B were then passed through a 1ml HisTrap (on the AKTA-Express) and eluted using a gradient elution. The elution fractions were analysed using SDS-PAGE electrophoresis; fractions containing ΔN-SNM1A or ΔC-SNM1B were combined and concentrated before loading on a S200 Superdex gel filtration column. Eluted fractions were analysed by SDS-PAGE electrophoresis. The fractions containing ΔN-SNM1A or ΔC-SNM1B were combined and concentrated to the desired concentration.

Real-time Fluorescence Assays: Real-time fluorescence assays were performed as described (3) using a 20-mer oligonucleotide (modified to contain BHQ1 on the nucleotide on its 5′ end and a fluorescein label 8 nucleotides away; Eurofins Genomics). Reactions were carried out in black 384-well microplates, and measurements were made using a SpectraMax M2e fluorescent plate reader in fluorescent top read mode, with SoftMaxPro software (Molecular Devices, Sunnyvale, CA, USA) to control the settings. Reactions were performed in a total volume of 15 µl in the nuclease buffer (20mM HEPES-KOH pH 7.5, 50mM KCl, 10mM $MgCl_2$, 0.5mM DTT, 0.05% (v/v) Triton-X, 0.1mg/ml BSA, 5% (v/v) glycerol) with varying concentrations (0, 0.01, 0.1, 1, 10, 50, 100, 500, 1000nM) of compound (for IC_{50} determinations) or DNA substrate (for kinetics assays (10, 25, 50, 100, 250, 500, 1000, 2500nM)), 0.242 nM ΔN-SNM1A or 0.4 nM SNM1B (where indicated). Each reaction was started by the addition of ΔN-SNM1A/B, and the fluorescein emission spectra measured (excitation at 495 nm, emission at 525 nm and cutoff at 515 nm) with six readings taken at 7 s intervals for 6 min. The fluorescence intensity of each well was plotted against time, and the rate of increase was determined, plotted against compound or substrate concentration and fitted to a log(inhibitor)-response or Michaelis–Menten curve on Prism software (GraphPad Software, Inc., La Jolla, CA, USA) to determine IC_{50} or Km and Vmax. Error bars were plotted from three independent repeats.

Radioactive Nuclease Assays: To measure exonuclease activity, ΔN-SNM1A (0.35 ng, 0.8 nM) or SNM1B (0.3ng, 0.8nM) and BcII MBL (2µM, if applicable) was mixed with 1 pmol (100 nM) of 3′ -[32P]-labeled DNA substrate, nuclease buffer (described above) and increasing concentration of inhibitor to a total volume of 10 µl. Reactions were incubated at 37°C for 20 minutes and stopped by adding 2 µl of 80% (v/v) formamide/ 10 mM EDTA to each reaction and heating at 95°C for 5 min. Following separation on a 20% polyacrylamide/ 7 M urea denaturing gel, substrate and product bands were visualized by a Typoon Trio+ Variable Model Imager (GE Healthcare Bio-Sciences, Pittsburgh, PA, USA).

MTT Assay: MTT (3-(4, 5-dimethylthiazolyl-2)-2,5-diphenyltetrazolium bromide) assays were carried out using clear 96-well microplates where 5000 cells/well of HeLa cells were seeded for at least 4 hours before they were treated with various concentrations (0-50µM) of cephalosporin inhibitor. The inhibitors were left on the cells for 72 hours before 100µl/well of 1mg/ml MTT (Sigma Aldrich, dissolved in PBS) was added. The cells were incubated with MTT for 2 hours at 37°C before it was removed and 100µl/well of DMSO was added. The plates were then shaken at room temperature for 20 minutes before the absorbance at 570nm of each well was measured using the SoftMaxPro software (Molecular Devices, Sunnyvale, CA, USA). To test for sensitization to the ICL inducer SJG-136 (Gregson et al. 2001; Hartley and Hochhauser 2012; Hopton and Thompson 2011), the MTT assay was used to determine if the IC_{50} of HeLa cells in SJG-136 was affected by the presence of a fixed concentration of cephalosporin. IC_{50}s were calculated using Prism software (GraphPad Software, Inc., La Jolla, CA, USA), fitting the data to a log(inhibitor) versus response curve.

NMR Experiments: All NMR spectra were acquired at 298 K using a Bruker AVIII 700 spectrometer with $^{1}H/^{13}C/^{15}N$ TCI cryoprobe.

For ^{1}H NMR experiments, the data were recorded employing pulse sequence with water suppression (excitation sculpting with gradients using perfect echo). Spectra were recorded with 11161 Hz sweep width, 2 s relaxation delay, 65536 data points and 8 scans. For data processing line broadening of 2 Hz was used. The NMR samples were prepared in 50 mM TRIS-d11 buffer pH 7.5, supplemented with 10% D_2O.

Typical experimental parameters for Carr-Purcell-Meiboom-Gill (CPMG) NMR spectroscopy[20] were as follows: The PROJECT-CPMG sequence (90°x−[τ−180°y−τ−90°y−τ−180°y−τ]n−acq) was applied. Water suppression was achieved by pre-saturation. Data were collected with a sweep width of 11194 Hz and an acquisition time of 1.46 s. The filter width was 625000 s long and 64 scans were applied. Prior to Fourier transformation, the data were multiplied with an exponential function with a line broadening of 2 Hz.

In water-Ligand Observed Gradient SpectroscopY (wLOGSY)[18], the typical experimental parameters were as follows: number of scans 512., the data were collected with a sweep width of 11160 Hz, an acquisition time of 2.9 s, and a relaxation delay of 2 s. Prior to Fourier transformation, the data were multiplied with an exponentional function with a line broadening of 2 Hz.

Thermal shift assay Differential Scanning Fluorimetry assays were performed in white 8 x 6 PCR plates using a MiniOpticon™ Real-Time PCR Detection System (BioRad). Reactions were performed in 50 µl. Protein unfolding was monitored by measuring the fluorescence of the SYPRO Orange dye (Invitrogen). Temperature was increased from 25 to 85 °C, 1 °C increment every 30 seconds. The dye stock (5000x concentrate) was first diluted by adding 1 µl to 2.5 ml of 50 mM HEPES-NaOH pH 7.5, 200 mM NaCl metal free buffer to produce a 2x concentrated solution. Compounds were tested at 1 mM final concentration. The enzyme was diluted in SYPRO Orange buffer to obtain a final assay concentration of 6 µM. Measurements were performed in triplicate. For determination of T_M values, melting curves for each triplicate data set were analyzed using GraphPad Prism (GraphPad Software, Inc., La Jolla, CA, USA).

Supplementary Figure S1

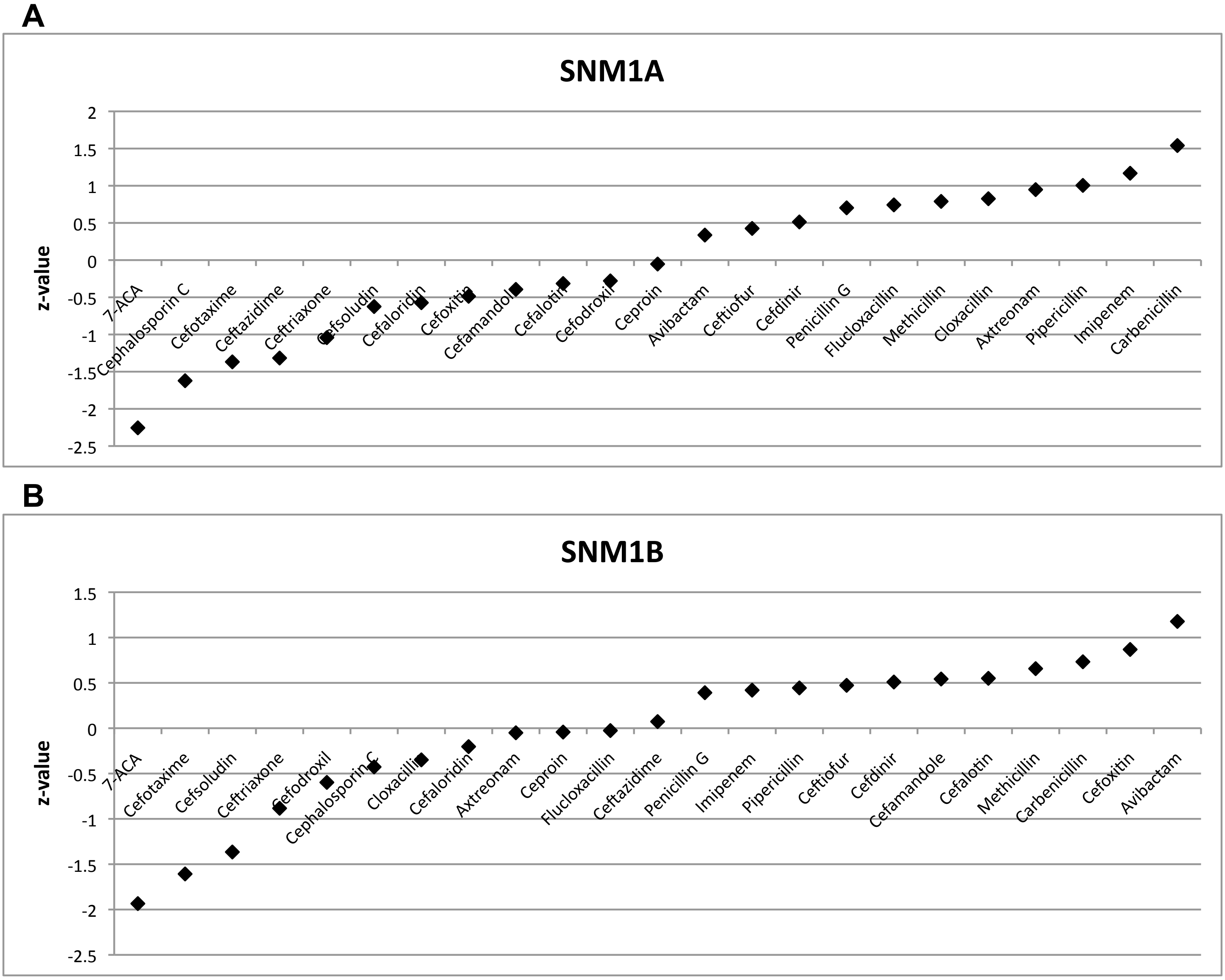

Supplementary Figure S1: Graphs showing z-values of β-lactam antibiotics against (A) SNM1A, and (B) SNM1B. Z-values were ranked from lowest to highest. Values are the average z-value from 3 independent repeats.

Supplementary Figure S2

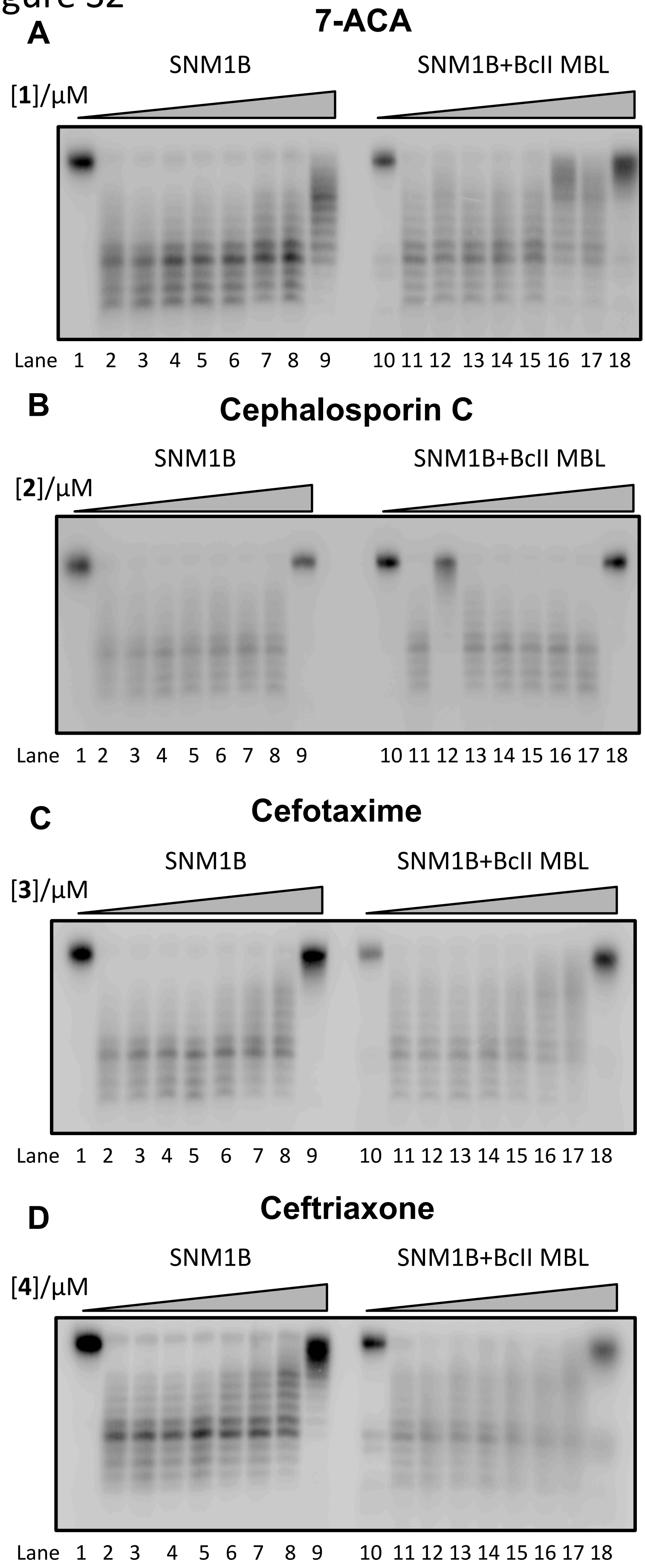

Supplementary Figure S2: Gel Images showing the effect of increasing concentration of (A) **1** (7-ACA), (B) **2** (Cephalosporin C), (C) **3** (Cefotaxime), and (D) **4** (Ceftriaxone) on the digestion of a 3'-radiolabelled 21 nucleotide DNA substrate (100nM) by SNM1B (0.8nM) in the presence (Lanes 10-18) and absence (Lanes 1-9) of 2μM BclI MBL.

Supplementary Figure S3

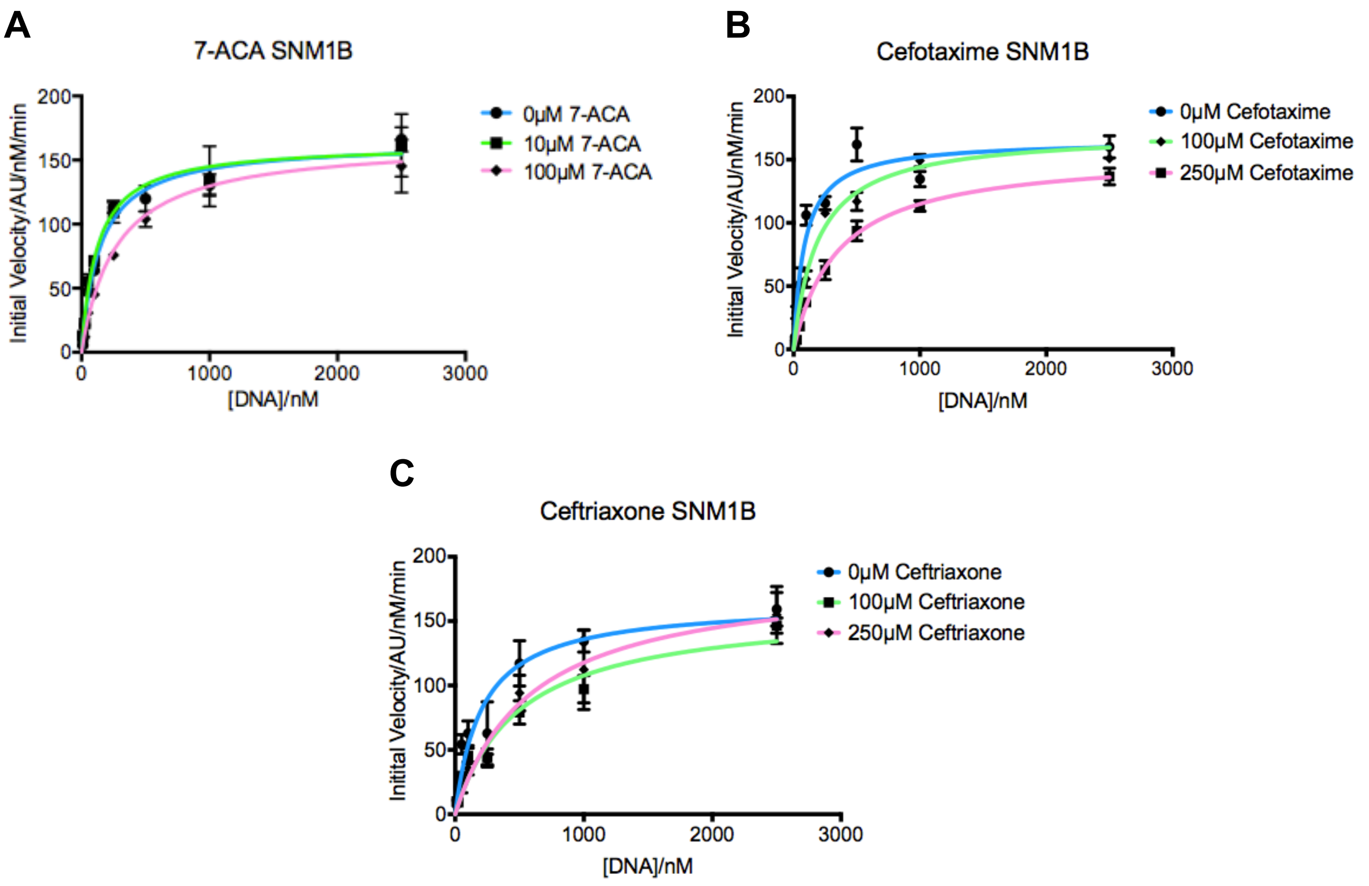

Supplementary Figure S3: Graphs showing Michaelis-Menten curves of SNM1B in increasing concentrations of 7-ACA (A), cefotaxime (B) and ceftriaxone (C). Graphs fitted using Prism software and K_M and V_{max} values generated using these curves.

Supplementary Figure S4

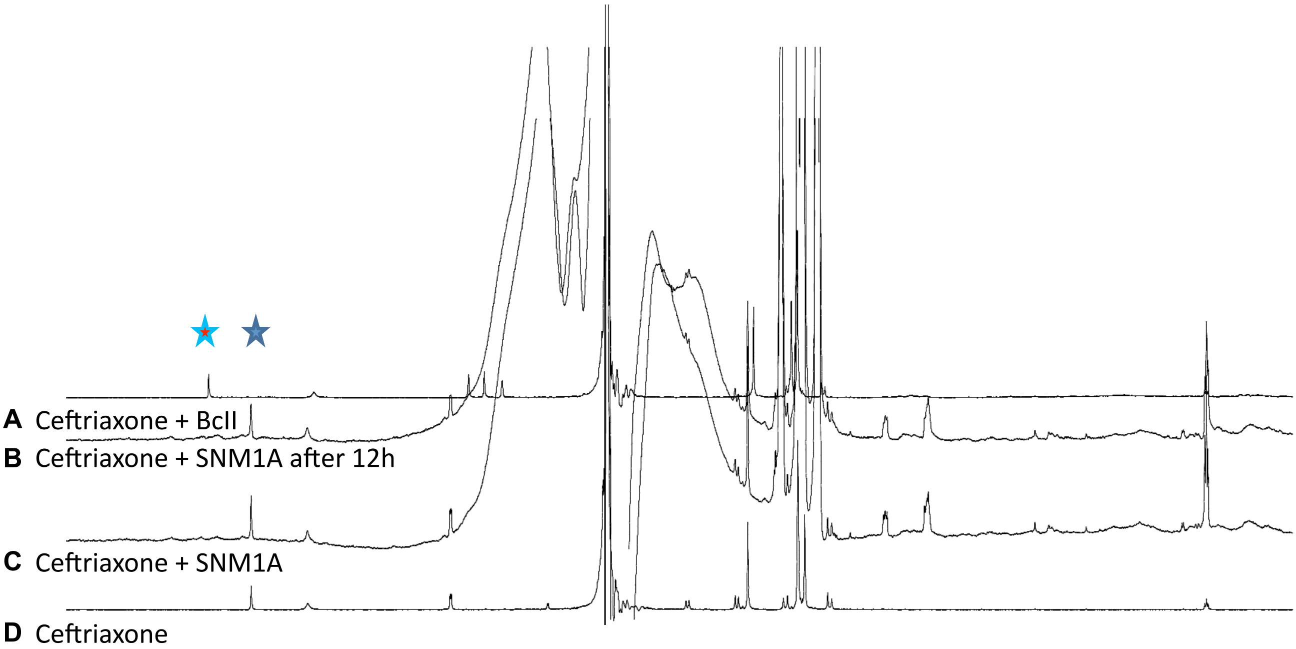

Supplementary Figure S4: NMR Spectra of ceftriaxone (A) after 10 minutes incubation with BcII MBL, (B) after 12h incubation with SNM1A, (C) after 10 minutes incubation with SNM1A, and (D) ceftriaxone alone.

Supplementary Figure S5

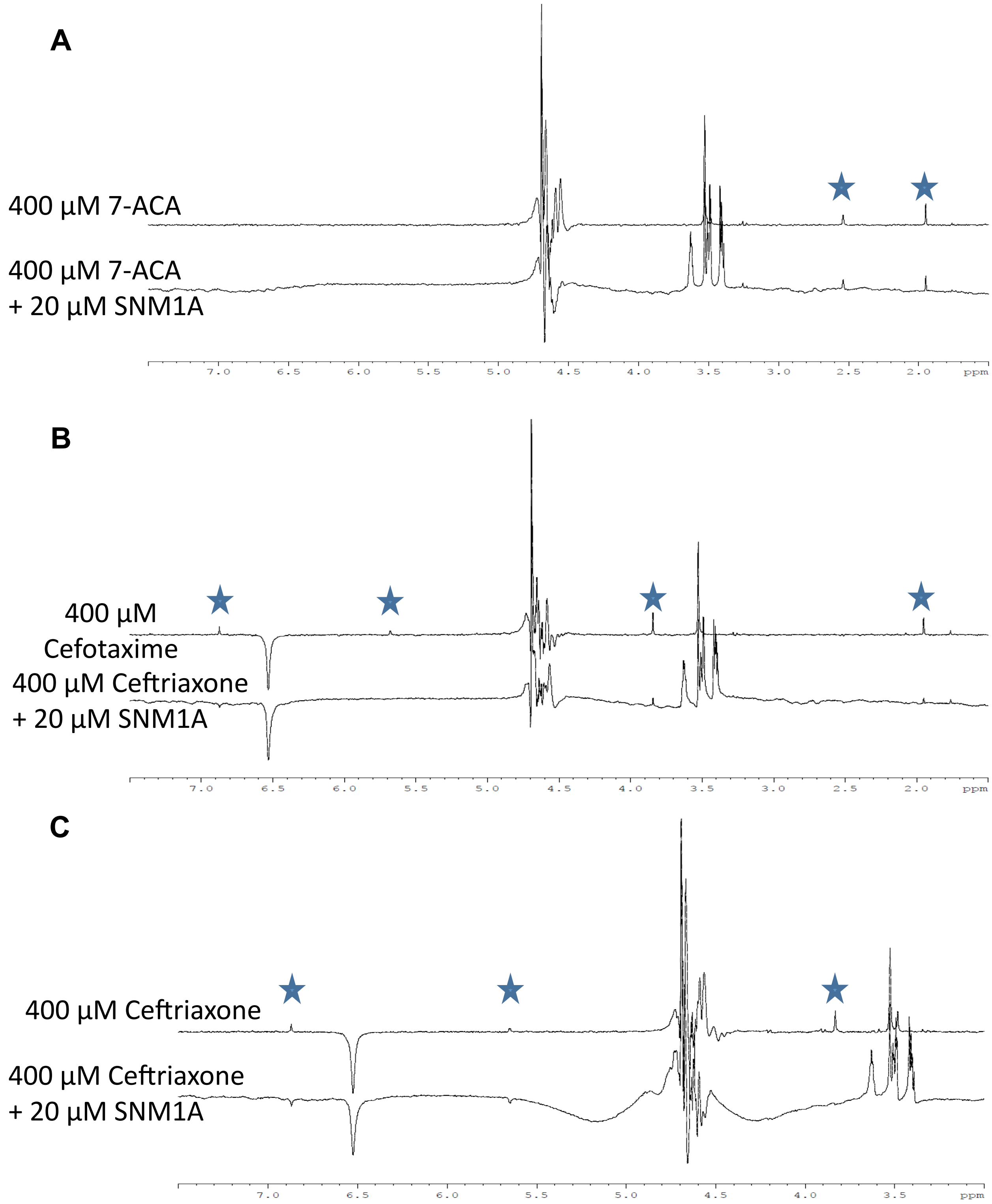

Supplementary Figure S5: waterLOGSY spectra of (A) 7-ACA, (B) cefotaxime and (C) ceftriaxone in the absence and prescence of 20µM of SNM1A.

Supplementary Figure S6

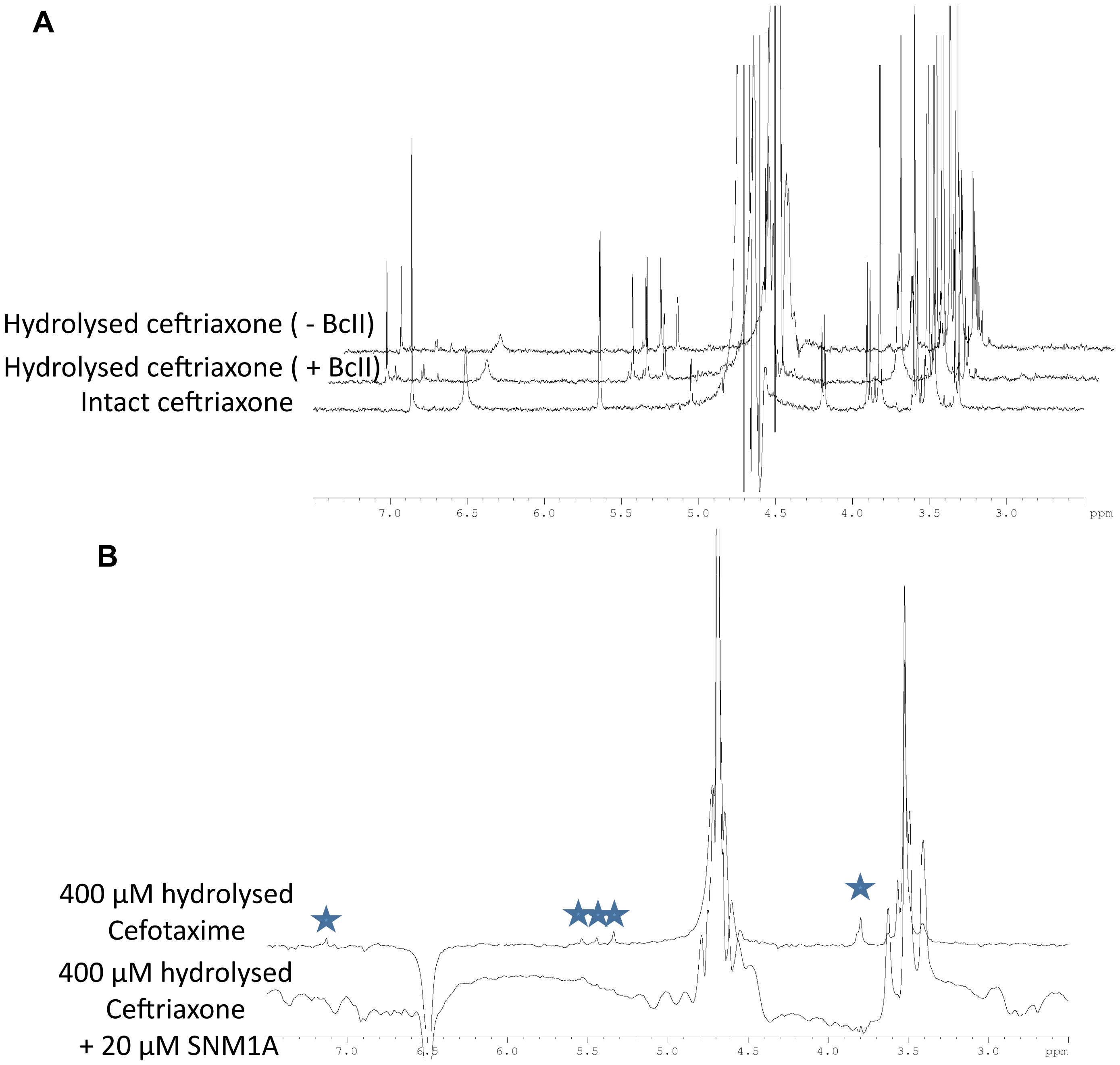

Supplementary Figure S6: (A) ^{1}H NMR spectra of intact ceftriaxone overlayed with the hydrolysed ceftriaxone with BcII and after 'semi-purification', without BcII. (B) waterLOGSY spectra of hydrolsed cefotaxime in the presence and absence of 20µM of SNM1A.

Supplementary Figure S7

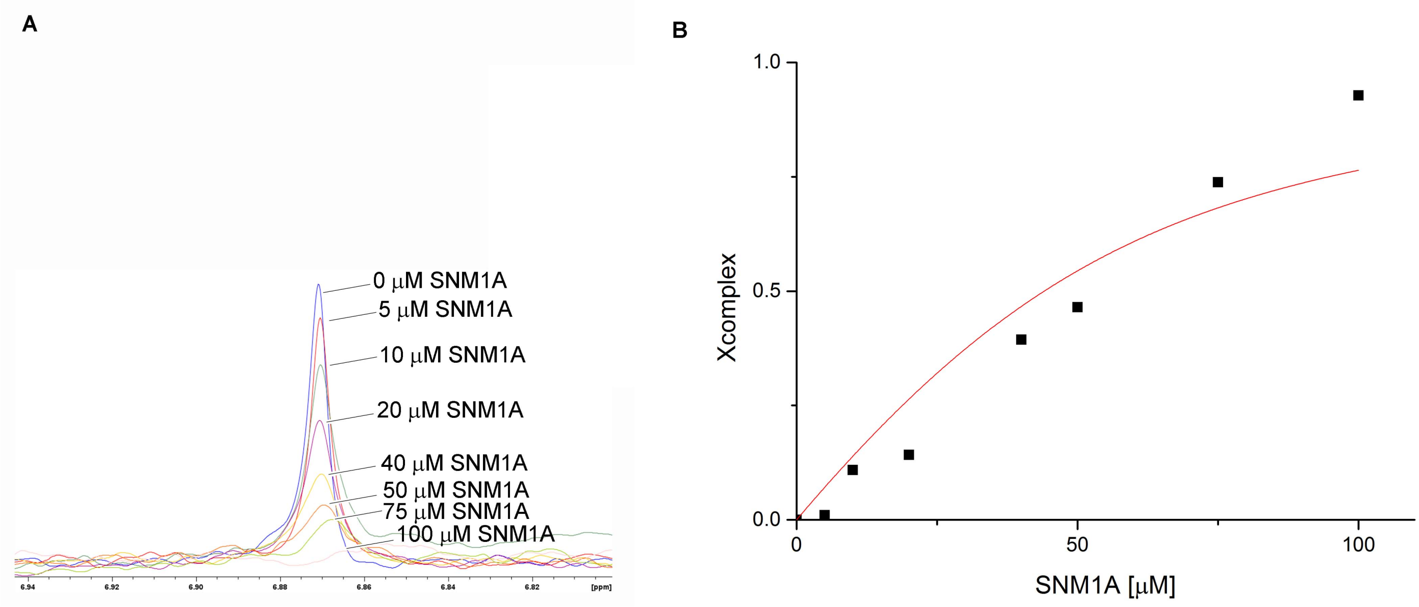

Supplementary Figure S7: (A) ^{1}H CPMG spectrum of ceftriaxone with increasing amounts of SNM1A. (B) Quantification from CPMG spectrum of ceftriaxone used to determine K_d of ceftriaxone with SNM1A.

Supplementary Figure S8

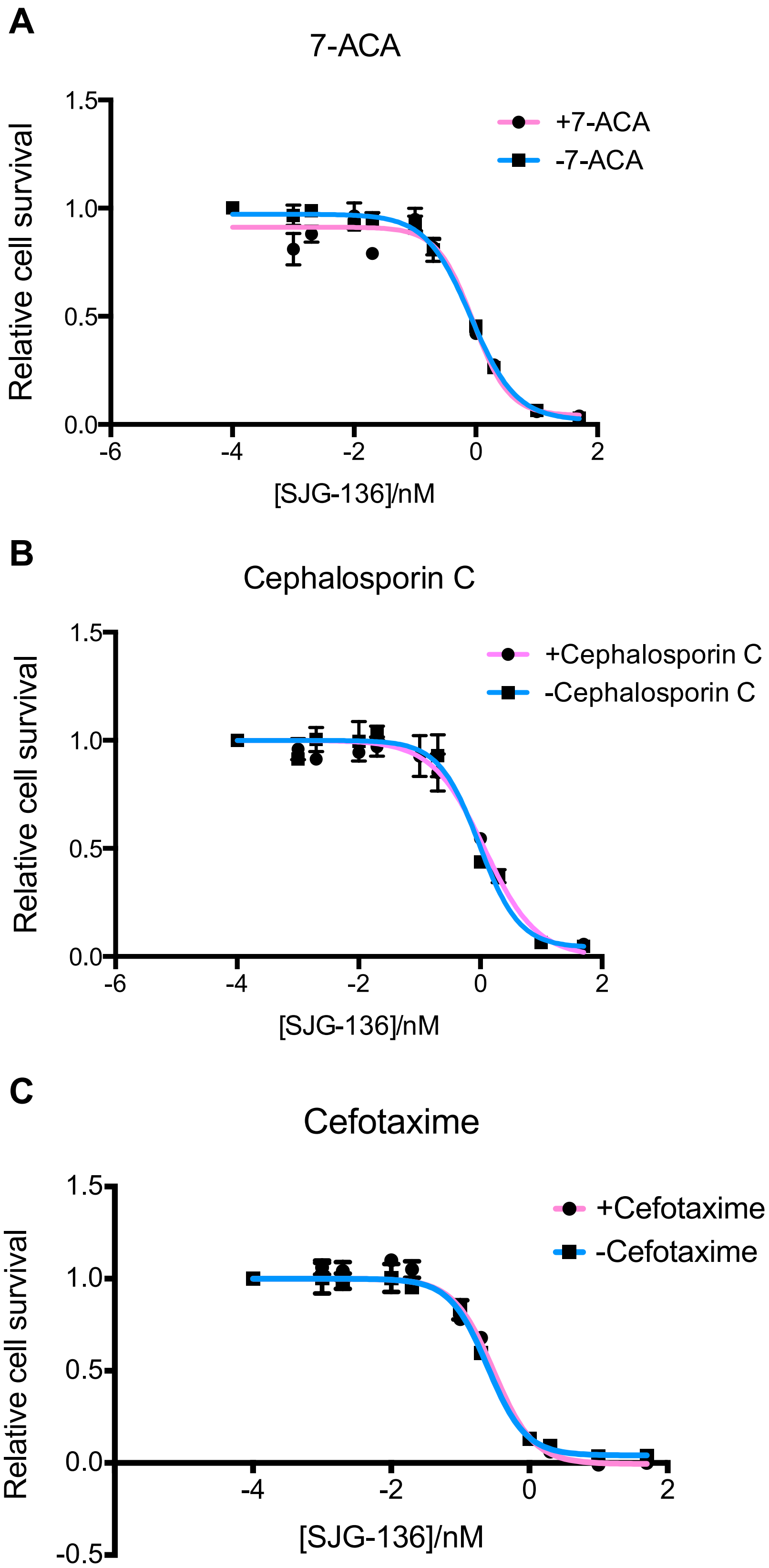

Supplementary Figure S8: Graphs showing survival of HeLa cells measured by MTT assays in increasing concentration of the ICL-inducing drug, SJG-136, in the presence and absence of (A) 7-ACA, (B) Cephalosporin C and (C) Cefotaxime. Cell survival represented relative to the DMSO treated control cells. Error bars represent standard error of the mean from 3 independent repeats.

Supplementary Table 1: Table showing list of cephalosporins tested.

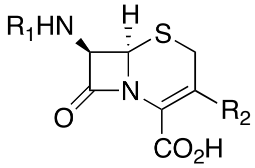

Name	R_1	R_2	IC_{50} SNM1A/μM	IC_{50} SNM1B/μM
Cefadroxil	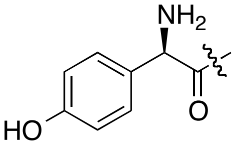	$-CH_3$	>500	>500
Cephalaxin	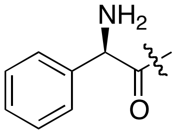	$-CH_3$	>500	>500
7-ACA	H	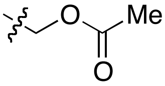	7 ± 1	32 ± 10
Cephalosporin C	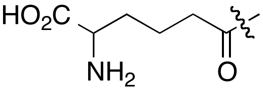	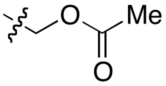	61±27	>500
Cefalotin	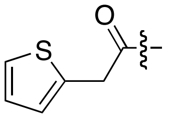	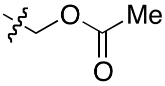	>500	>500
Cefoxitin	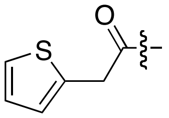	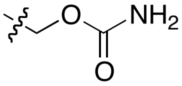	>500	>500
Cefaloridin	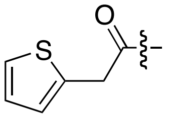	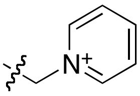	>500	>500
Cefotaxime	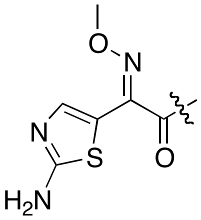	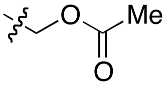	5 ± 2	129 ± 20
Cefdinir	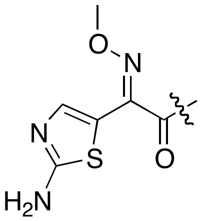	$-CH=CH_3$	>500	>500
Ceftazidime	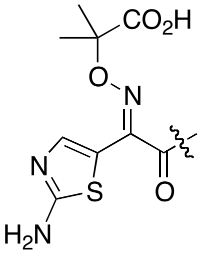	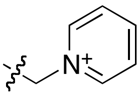	>500	>500

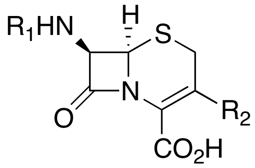

Name	R_1	R_2	IC_{50} SNM1A/µM	IC_{50} SNM1B/µM
Ceftiofur	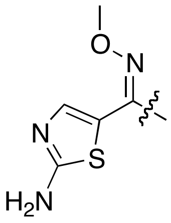	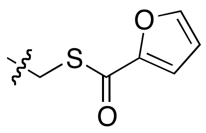	>500	>500
Ceftriaxone	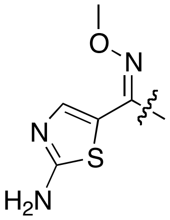	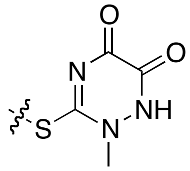	4 ± 2	40 ± 14
Cefamandole	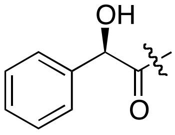	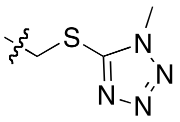	>500	>500
Cefsulodin	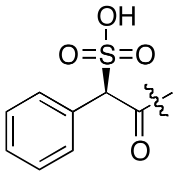	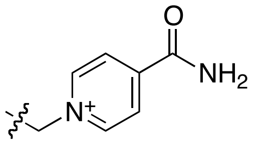	>500	>500

Supplementary Table 2: Table showing list of Penicillins tested.

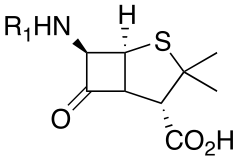

Name	R_1	IC_{50} SNM1A/µM	IC_{50} SNM1B/µM
Penicillin G	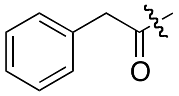	>500	>500
Carbenicillin	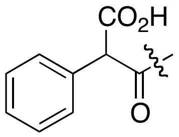	>500	>500
Methicillin	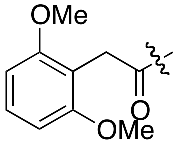	>500	>500
Cloxacillin	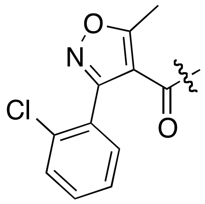	>500	>500
Flucloxacillin	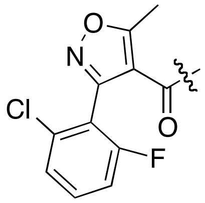	>500	>500
Piperacillin	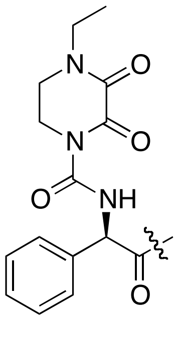	>500	>500

Supplementary Table 3: Table showing list of other antibiotics tested.

Name	Strucutre	IC_{50} SNM1A/µM	IC_{50} SNM1B/µM
Aztreonam	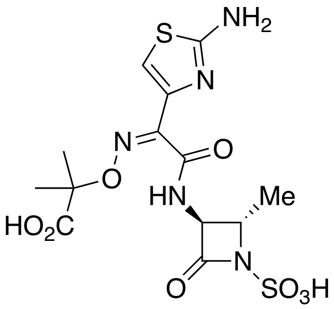	>500	>500
Imipenem	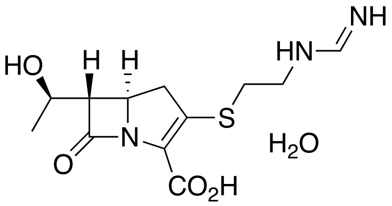	>500	>500
Avibactam	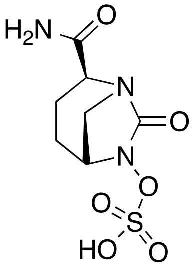	>500	>500
